# Vapor Pressure and Evaporation Studies of Saline Solutions on Natural and Synthetic Fabrics for Industrial Water Treatment

**DOI:** 10.3390/polym16162335

**Published:** 2024-08-18

**Authors:** Alexis López-Borrell, Jaime Lora-García, Salvador C. Cardona, María-Fernanda López-Pérez, Vicent Fombuena

**Affiliations:** 1 Instituto de Seguridad Industrial, Radiofísica y Medioambiental (ISIRYM), Universitat Politècnica de València (UPV), Plaza Ferrándiz y Carbonell, s/n, 03801 Alcoy, Spain; jlora@iqn.upv.es (J.L.-G.); scardona@iqn.upv.es (S.C.C.); malope1@iqn.upv.es (M.-F.L.-P.); 2 Technological Institute of Materials (ITM), Universitat Politècnica de València (UPV), Plaza Ferrándiz y Carbonell 1, 03801 Alcoy, Spain; vifombor@upv.es

**Keywords:** vapor pressure, evaporation, natural fibers, absorption, relative humidity

## Abstract

In the present paper, we have conducted a comprehensive analysis of vapor pressures of both saturated and unsaturated solutions, alongside a study of evaporation using synthetic and natural fabrics for industrial applications in brackish water treatment under zero liquid discharge (ZLD) philosophy. By determining the vapor pressures of saturated solutions, we obtained results consistent with those of other researchers, extending the range of tested temperatures from 1 to 50 °C and successfully fitting the parameters of an Antoine-type equation. Similarly, positive results were achieved for unsaturated solutions, where various parameters of different equations accounting for the salt concentration were estimated, simplifying the fitting procedure. Natural evaporation tests from water surfaces using saturated solutions revealed that salts with higher associated vapor pressures exhibit higher evaporation rates. On the other hand, hydrated salts retain water in their structure and are significantly affected by ambient humidity. Evaporation studies on natural and synthetic fabrics with saturated NaCl and CuSO_4_·5H_2_O solutions showed distinct behaviors. NaCl increased both the evaporation rate and salt deposition with each cycle. In contrast, CuSO_4_·5H_2_O reduced the absorption capacity by blocking the fabric’s structure, decreasing the evaporation efficiency over successive cycles.

## 1. Introduction

Demand for water resources has become a significant issue in many parts of the world, even in regions where it was not previously a concern. The decline in water availability is primarily due to the misuse and mismanagement of water resources and water pollution. These challenges are further exacerbated by population growth, increasing demand for this essential resource and leading to overexploitation [1].

Much of the research over the years has focused on investigating water deterioration, the main pollutants, and their origin. A large part of water pollution is a consequence of industrial activity and the pollution from urban areas. Water pollution due to industrial activity is diverse, and it ranges from mining activity with heavy metal pollution, pesticide pollution and fertilizer leaks from the agri-food industry to companies that generate untreated excess water discharges [2,3,4]. On the other hand, a significant source of pollution is seen in urban areas due to the large amount of water consumed per inhabitant. It has been observed that water quality has been worsening over time, as it is not treated correctly, causing an impact on human health [5,6,7,8].

Conventional treatments mainly used for wastewater management involve physicochemical processes such as aerobic and anaerobic biological processes used in wastewater treatment plants, chemical coagulation-flocculation processes and the combination of physical techniques such as membrane separation treatment. More advanced techniques based on membrane separation include microfiltration (MF), ultrafiltration (UF), nanofiltration (NF) or reverse osmosis (RO) in conjunction with the other conventional processes for higher separation efficiency or to ensure the quality of the wastewater to be treated [9,10]. On the other hand, one of the working philosophies for wastewater treatment lies in Zero Liquid Discharge (ZLD) [11]. This method does not recover water from its treatment but concentrates the waste in the wastewater. Afterwards, it can be treated to either form a highly concentrated liquid or to leave a solid residue by removing the remaining liquid [12,13,14]. Also, in this case, depending on the type of industry operating under ZLD, it may be beneficial to revalue the waste and create a circular economy within the industry.

The ZLD methodology has been used for many years to reduce the volume of waste and concentrate it to very high levels. One of the techniques generally used is evaporation processes, either by forced convection, which requires high energy consumption, or by natural evaporation, which requires typically high evaporation surfaces such as salt ponds [15]. Due to the two main disadvantages of evaporation techniques—high energy consumption and the need for large working surfaces—a viable alternative that allows evaporation processes to operate with lower energy consumption and smaller working surfaces must be found. An alternative approach is to use parallel sheets of material immersed in the problem liquid and then exposed to air for drying. This method increases the available evaporation surface area by arranging multiple parallel sheets in a treatment bath [16].

In recent years, the production of natural fabrics has increased, as they are seen as an alternative to conventional synthetic fabrics such as glass fiber, carbon fiber or aramid to manufacture composite materials. These natural fabrics can compete with synthetics due to their low price and acceptable mechanical properties. Some fabrics or natural fibers most commonly used and with a high annual production include jute, bamboo or flax, among many others [17,18,19]. Bamboo fibers have a yearly production of 30 Mt, Jute fibers 2.3 Mt, and Flax fibers 0.83 Mt [20,21,22]. These natural fibers exhibit significant annual production rates, with even more growth expected in the coming years.

Natural and synthetic fabrics have versatile applications beyond composite materials. Their structure enables water and contaminants to be retained within, with liquid absorption capacities ranging from 100 to 850% of their initial weight [23]. The water absorption property within their structure facilitates their alignment in parallel within a treatment bath for evaporation processes. This reduces the energy consumption and required surface area and offers scalability due to its ease of implementation. An example of the versatility that natural fabrics can offer for these objectives is the article authored by the researchers, in which a comprehensive characterization of the fabrics is conducted from a physicochemical, mechanical, and absorbent capacity perspective [23]. However, the evaporation capacity when applying a technology based on the ZLD philosophy does not solely depend on the type of fabric. For instance, it is very important to determine the influence of the types of salts present in brackish water and to study the vapor pressure, as recent studies have shown [24].

To enhance the natural evaporation rate, the idea of combining absorbent surfaces, such as natural and synthetic fabrics, to increase the available evaporation surface by leveraging wind currents is explored. This technique, known as Wind-Aided Intensified eVaporation (WAIV), does not recover water; instead, it facilitates its evaporation into the environment. However, it can be employed to diminish waste volume or recover mineral salts in the water, which precipitate on the absorbent fabrics during the process [25,26].

This study investigates the behavior of various salts in a saturated state and at different concentrations to determine their vapor pressure across a wide range of temperatures. Additionally, it aims to assess the evaporation rate of these solutions on different surfaces, including natural and synthetic fabrics, along various operational cycles.

## 2. Materials and Methods

### 2.1. Materials

#### 2.1.1. Fabrics

Six fabrics pre-selected from previous research were employed to carry out the tests during this study [23]. This selection encompasses both natural and synthetic fabrics. On the one hand, natural fabrics such as jute (Jut), bamboo (Bam), linen composed of 50% polylactic acid (LPLA) and, finally, a non-woven fabric composed of 70% palm prunings, 20% lyocell and 10% PLA (WL-T), were included. The WL-T fabric was supplied by AITEX (Alcoy, Spain). Additionally, two synthetic fabrics were employed: a non-woven polyester fabric (PES) and a fabric with an aramid taffeta structure (Ara). Both of these fabrics were supplied by Castro Composites (Pontevedra, Spain).

#### 2.1.2. Salts

For the experiments, salts include sodium nitrate (NaNO_3_) supplied by Scharlau Chemie S.A. (Barcelona, Spain), and calcium hydroxide (Ca(OH)_2_), potassium nitrate (KNO_3_), sodium hydrogen carbonate (NaHCO_3_), magnesium sulphate (MgSO_4_·7H_2_O), and copper sulphate (CuSO_4_·5H_2_O) provided by PanReac Applichem, ITW Reagents (Barcelona, Spain). Additionally, sodium chloride (NaCl) delivered by Carlo Erba Reagents S.A. (Barcelona, Spain) was tested. All these salts were used for vapor pressure determination tests and the natural evaporation of saturated solutions using natural and synthetic fabrics.

These salts were selected because they were used in previous research to synthesize brackish water [23]. Additionally, NaCl was chosen for further study because it is the most abundant salt in seawater and may present an alternative treatment pathway.

### 2.2. Experimental Methodology

#### 2.2.1. Natural Evaporation of Saturated Salts

Saturated solutions of the salts mentioned above were prepared for the evaporation studies of concentrated salts. Natural evaporation tests were conducted for each salt using three beakers, each with a different evaporation surface, to assess its effect on the evaporation rates of water. The evaporation surfaces utilized are outlined in Table 1.

The tests involved placing a quantity of the saturated solution in different beakers and recording the initial mass of the solution using an analytical balance, Nahita Blue Series 5134. The analytical balance has a maximum capacity of 220 g, with a resolution of 10 mg and a weighing accuracy of ±1 mg. Mass measurements were taken every 24 h for each sample to record the water loss due to natural evaporation. These tests continued until the mass between two consecutive 24-h measurements remained unchanged.

#### 2.2.2. Vapor Pressure Experiments for Saturated Salts

**Figure 1:**
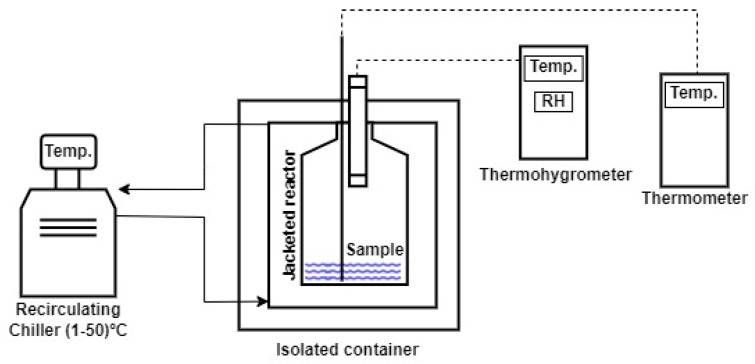
Experimental set-up for the determination of vapor pressures.

The vapor pressure was determined for the seven salts mentioned above in their saturated form. Saturated solutions were prepared in a volume of 75 mL and placed in a hermetically sealed jacketed reactor to control the temperature of the liquid and air using a Lauda Ecoline RE104 Recirculating Chiller, supplied by Lauda DR. R. Wobser GMBH & CO. KG (Lauda-Königshofen, Germany). These vapor pressure tests were conducted over a temperature range of 1 to 50 °C, in increments of 5 °C. The experimental set-up is illustrated in Figure 1.

The relative humidity (*RH*) measurement was used to determine the vapor pressure. For *RH* measurements, a Testo 635-2 thermohygrometer was used (Instrumentos Testo S.A., Barcelona, Spain), with connectable temperature and humidity probes inserted into the hermetically sealed container and placed in the upper gas chamber. This probe has a temperature measurement range of −20 to 70 °C with an accuracy of ±0.3 °C. Additionally, the humidity measurement range is 0 to 100% with an accuracy of ±2%. The temperatures were adjusted using the recirculation chiller. The *RH* was measured 30 min after reaching the temperature setpoint to ensure equilibrium with the air. An additional flexible Pt-100 temperature probe (Instrumentos Testo S.A., Barcelona, Spain) was used to check the temperature of the liquid phase. This flexible temperature probe has a measurement range of −80 to 300 °C with an accuracy of ±0.05 °C.

For the determination of the vapor pressure, Equation (1), which relates to relative humidity, is used:

(1) $$RH\;\left(\%\right)=\frac{P_{v}}{P_{v,sat}}\cdot 100$$

where *RH* is the relative humidity measured in %, *P_v_* is the vapor pressure of the solution in Pa and *P_v,sat_* is the saturation vapor pressure of pure water in Pa. The variable *P_v,sat_* (Pa) is obtained from Equation (2) based on the IAPWS datasheets, but improving the fitting errors [27]:

(2) $$P_{v,sat}=\frac{\exp\left(34.494-\frac{4924.99}{T+237.1}\right)}{{\left(T+105\right)}^{1.57}}$$

where *T* is the temperature, measured in °C. Note that this expression is valid only for temperatures above 0 °C.

#### 2.2.3. Vapor Pressure Experiments for Unsaturated Salts

NaCl and CuSO_4_·5H_2_O salts were used in different molal concentrations in the temperature range from 1 to 50 °C, in 5 °C increments, for the vapor pressure tests of unsaturated solutions. The molalities chosen for the tests were 0.08, 0.17, 0.60, 1.23, 2.71, 3.68 and 5.75 mol·kg^−1^. The vapor pressure was determined in the same way as for the tests with saturated salts using the Testo 635-2 thermohygrometer to measure the relative humidity of the air once equilibrium had been reached, according to the experimental set-up in Figure 1.

#### 2.2.4. Evaporation on Natural and Synthetic Fabrics

Saturated solutions of NaCl and CuSO_4_·5H_2_O salts were used for evaporation tests on natural and synthetic fabrics. These experimental tests were conducted under laboratory conditions, with the room maintained at a temperature of 20 ± 2 °C and a relative humidity of 40 ± 5%.

**Figure 2:**
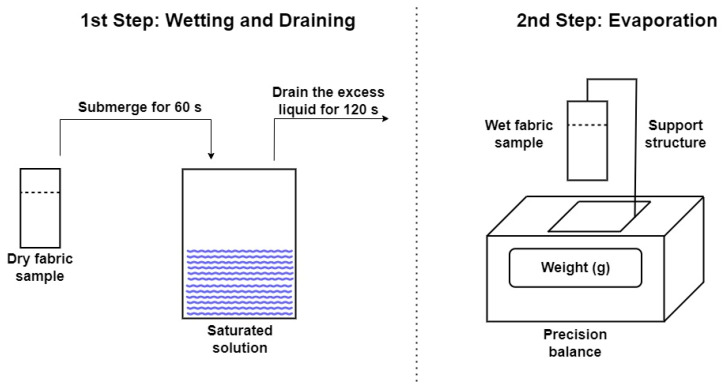
Experimental set-up for the evaporation experiments.

The fabrics used for the evaporation tests had a surface area of 50 mm × 75 mm. To control the evaporation surface, the wet area will be confined to 50 mm × 60 mm, leaving the remaining surface available for handling the fabric. Two samples of each fabric with this surface area were prepared, and the effects of evaporation during wet and dry operation cycles were studied for each salt. To wet the samples they were inserted obliquely into the solution and kept immersed for (60 ± 1) s, then removed from the solution and allowed to drain the excess liquid for (120 ± 3) s, as shown in the first step of Figure 2. After this time, the samples were placed on a support and measurements of water weight loss were taken using an analytical balance (Nahita blue series 5134), as shown in the second step of Figure 2. The test continued until the fabric was completely dry.

Multiple evaporation cycles were performed to study the evaporation of the saturated solutions on the fabrics. Each fabric underwent 15 wetting and drying cycles for each solution studied. The evaporation rates for cycles 1 (1C), 2 (2C), 5 (5C), and 15 (15C) were compared to observe the effect of salt precipitation on the fabrics. This approach aims to examine the impact of salt accumulation on evaporation rates and also the amount of salt deposited on the fabrics.

### 2.3. Parameter Fitting for Different Expressions of the Vapor Pressure of Saturated and Unsaturated Solutions

#### 2.3.1. Fitting the Parameters of the Antoine Equation to the Experimental Vapor Pressures of Saturated Solutions

The Antoine equation has been used [28], with its parameters *A*, *B*, and *C*, as shown in Equation (3), fitted to the experimental vapor pressures measured for the seven saturated salt solutions at different temperatures:

(3) $$\ln\left(P_{v}\right)=A-\frac{B}{C+T}$$

where *P_v_* is the vapor pressure of the saturated salt solutions in kPa, and *T* is the temperature in K.

#### 2.3.2. Fitting the Parameters to the Experimental Vapor Pressures of Unsaturated Solutions

A literature review of the various expressions used to determine the vapor pressure of unsaturated solutions reveals that most of them are based on polynomial equations. These equations consider both the solution’s concentration and its temperature dependence.

In this research, three fitting equations have been selected for testing. The first one is the Antoine equation (Equation (3)), where parameter estimations have been performed separately for each concentration, on the one hand, and the average, on the other hand. These fittings aim to determine if a simple expression can accurately predict the vapor pressures. The second and third equations are based on polynomial equations.

The equation described by Patil et al. [29] has three fitting parameters, as shown in Equation (4).

(4) $$\log\left(P_{v}\right)=A\left(m\right)+\frac{B\left(m\right)}{T}+\frac{C\left(m\right)}{T^{2}}$$

where *P_v_* is the vapor pressure in kPa, *T* is the temperature expressed in K and *A*, *B* and *C* are the fitting parameters that depend on the molality of the solution. This relationship is formulated through Equations (5), (6) and (7), respectively:

(5) $$A\left(m\right)=A_{0}+A_{1}\cdot m+A_{2}\cdot m^{2}+A_{3}\cdot m^{3}$$

(6) $$B\left(m\right)=B_{0}+B_{1}\cdot m+B_{2}\cdot m^{2}+B_{3}\cdot m^{3}$$

(7) $$C\left(m\right)=C_{0}+C_{1}\cdot m+C_{2}\cdot m^{2}+C_{3}\cdot m^{3}$$

where *m* is the molality of the solution expressed in mol·kg^−1^ and *A_i_, B_i_* and *C_i_* represent the respective fitting parameters. To simplify the optimization procedure, the objective is to minimize the total number of fitting parameters while ensuring that the final equation accurately represents the observed reality.

On the other hand, the following expression to be tested to explain the behaviour of unsaturated solutions at different concentrations is the one described by Shibue [30], as shown in Equation (8).

(8) $$\ln\left(P_{v}\right)=\ln\left(P_{c}\right)+g\left(T\right)+h\left(x\right)$$

where *P_v_* is the vapor pressure expressed in MPa, *P_c_* is the critical pressure expressed in MPa, *g(T)* is a temperature-dependent expression expressed in K and, finally, *h(x)* is a mole fraction-dependent expression. *P_c_* is obtained from Equation (9):

(9) $$P_{c}=22.064+q_{5}\cdot x+q_{6}\cdot x^{2}+q_{7}\cdot x^{3}+q_{8}\cdot x^{4}+q_{9}\cdot x^{5}+q_{10}\cdot x^{6}$$

where *x* is the experimental mole fraction of the salt used and the parameters *q_i_* are fitted values for a particular salt. In this work, the values of *q_i_* reported by Shibue for the H_2_O + NaCl mixture are assumed, as he stated these are well-studied and validated *P_c_* values (Table 2).

The function *g(T)* is obtained from the Equation (10). The author found that with this simplified version, the fits were accurate. The critical pressure of pure water is considered at 647.096 K, so the expression only depends on the experimental temperature values.

(10) $$\begin{array}{c} g\left(T\right)=\frac{647.096}{T}\cdot \left[-7.85951783\cdot \left\{1-\left(\frac{T}{647.096}\right)\right\}+1.184408256\cdot {\left\{1-\left(\frac{T}{647.096}\right)\right\}}^{1.5}\right]\\ +\frac{647.096}{T}\cdot \left[-11.7866497\cdot {\left\{1-\left(\frac{T}{647.096}\right)\right\}}^{3}+22.6807411\cdot {\left\{1-\left(\frac{T}{647.096}\right)\right\}}^{3.5}\right]\\ +\frac{647.096}{T}\cdot \left[-15.9618719\cdot {\left\{1-\left(\frac{T}{647.096}\right)\right\}}^{4}+1.80122502\cdot {\left\{1-\left(\frac{T}{647.096}\right)\right\}}^{7.5}\right] \end{array}$$

Finally, the function *h(x)* depends on the mole fraction (concentration) of the salt used and is obtained from Equation (11) for the dilute region, with a mole fraction ranging from 0 to 0.024. For the concentrated region, it is derived from Equation (12), for a mole fraction ranging from 0.024 to 0.117.

(11) $$h\left(x\right)=\frac{a_{2}\cdot x}{x+a_{1}^{2}}+a_{3}\cdot x^{2}$$

(12) $$\begin{array}{c} h\left(x\right)=\left[\frac{a_{1}^{2}\cdot a_{2}}{{\left(u+a_{1}^{2}\right)}^{2}}+2\cdot a_{3}\cdot u\right]\cdot \left(x-u\right)+b_{1}\cdot {\left(x-u\right)}^{2}\\ +b_{2}\cdot \left(x-u\right)\cdot \left(x^{2}-u^{2}\right)+b_{2}\cdot \left(x-u\right)\cdot \left(x^{2}-u^{2}\right)+\frac{a_{2}\cdot u}{u+a_{1}^{2}}+a_{3}\cdot u^{2} \end{array}$$

where *x* is the mole fraction, *u* is the value of the mole fraction where the change from the dilute to the concentrated region takes place and *a_i_* and *b_i_* are the fitting parameters for both expressions. Equation (11) is fitted first and then extended to Equation (12).

All parameter settings of the different equations were fitted using Matlab 2021a and the lsqnonlin function. A residual value, calculated as the sum of the squared differences between the experimental and theoretical data, was obtained to assess the quality of the fitting parameters. Additionally, corresponding confidence intervals were provided.

## 3. Results and Discussion

### 3.1. Vapor Pressure of Saturated Salt Solutions

**Figure 3:**
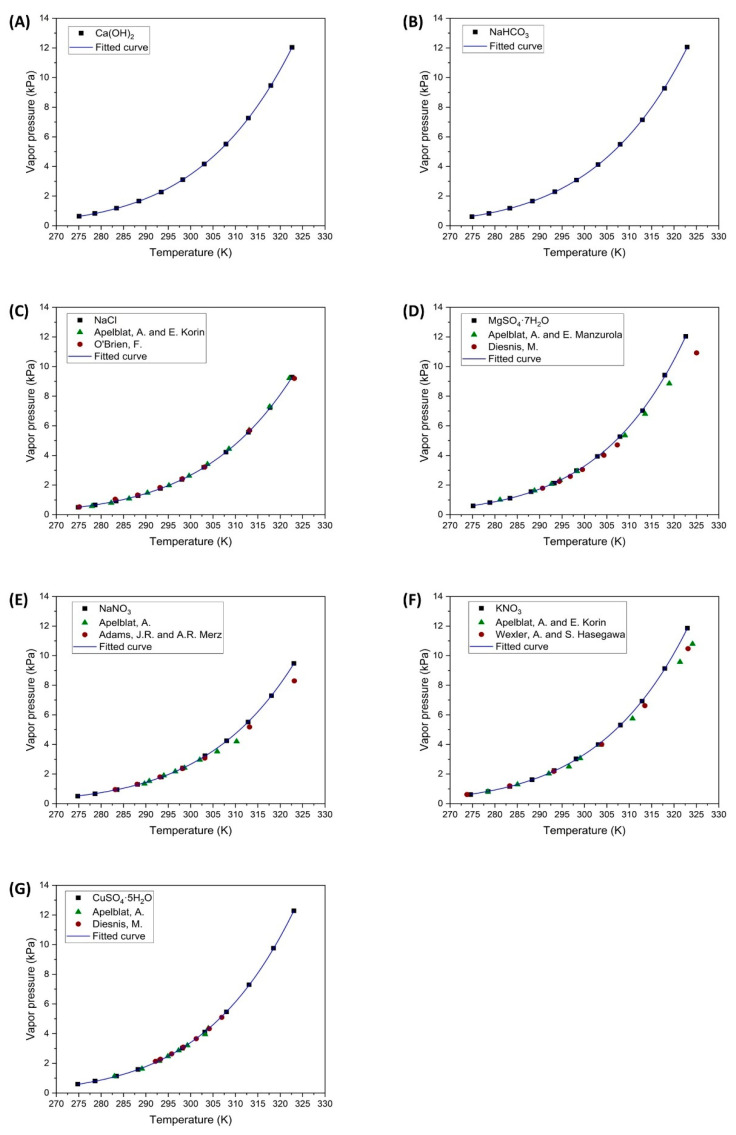
Vapor pressures of saturated salt solutions. (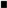 Experimental data; 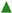 Apelblat, et al. [31,32,33,34,35]; 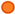 O’Brien, F. [36], Diesnis, M. [37], Adams, J.R. and A.R. Merz [38], Wexler, A. and S. Hasegawa [39]; 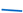 Antoine fitting curve).

The results of the determination of vapor pressures for saturated salt solutions are shown in Figure 3, and the values of the fitted parameters of Equation (3) can be seen in Table 3.

As shown in Figure 3, the experimental results obtained in this study align closely with those reported in the literature by other researchers who have determined the vapor pressures of saturated salt solutions.

The authors have observed that the application of Equation (2) results in values very similar to those obtained in the previous literature, as cited in the following references. These highly accurate results have been obtained with the salts Ca(OH)_2_ and NaHCO_3_. On the other hand, some salts do not show as much similarity with previously published results. These errors, ranging from 3% to 8%, are observed in the samples of MgSO_4_·7H_2_O, NaNO_3_, and KNO_3_ salts in Figure 3D–F, respectively. These differences in vapor pressure measurements become more pronounced at temperatures above 35 °C. In this study, the vapor pressures determined are slightly higher than those reported by other authors. These discrepancies could be attributed to variations in the experimental equipment used or the determination of *P_v,sat_* as shown in Equation (2). Despite these deviations, the values determined are considered accurate.

The vapor pressures of Ca(OH)_2_ and NaHCO_3_ solutions have been poorly studied. The saturated NaHCO_3_ solution exhibits high solubility within the temperature range investigated in this study, as noted by Ozcan and Miller [40]. Leon-Hidalgo et al. [41] determined the vapor pressure of a saturated sodium bicarbonate solution using an indirect hygrometer measurement at 25 °C, obtaining a value of 3.18 kPa. The present work has recorded a vapor pressure value of 3.08 kPa at 25.1 °C, closely matching Leon-Hidalgo’s results. Knuutila et al. [42] investigated the sodium bicarbonate solution at the equilibrium point where sodium carbonate (Na_2_CO_3_) forms without reaching saturation. The values across the different concentration ranges are similar to those obtained for the saturated NaHCO_3_ solution.

Finally, the Ca(OH)_2_ solution has been scarcely studied due to its low solubility in water within the working temperature range. Duchesne and Reardon [43] compiled data from other authors indicating that the solubility of the Ca^2+^ ion was around 0.02 m, thus highlighting its low solubility level. Other researchers have increased the solubility of this salt by using bases, such as NaOH, at high concentrations and 25 °C, as investigated by Pallagi et al. [44]. Another study by Konno et al. [45] explored the solubility of this salt with varying concentrations of NaOH and temperatures of 25, 50, and 75 °C. The present study observed that due to this low solubility, the saturated Ca(OH)_2_ solution exhibited relative humidity values above 95% at a temperature of 15 °C, indicating that its vapor pressure closely approximates that of pure water as the temperature increases.

The parameter fitting of Equation (3), corresponding to Antoine’s equation, has demonstrated a good overlap with the experimental data, resulting in a low fitting error for all the salt solutions tested. Leon-Hidalgo et al., [41] conducted studies with both saturated and unsaturated saline solutions, fitting the saturated solutions to an Antoine-type expression. The parameters that were fitted in their study are similar to those obtained in the present research.

### 3.2. Vapor Pressure of Unsatured Solutions

#### 3.2.1. NaCl Solutions

**Figure 4:**
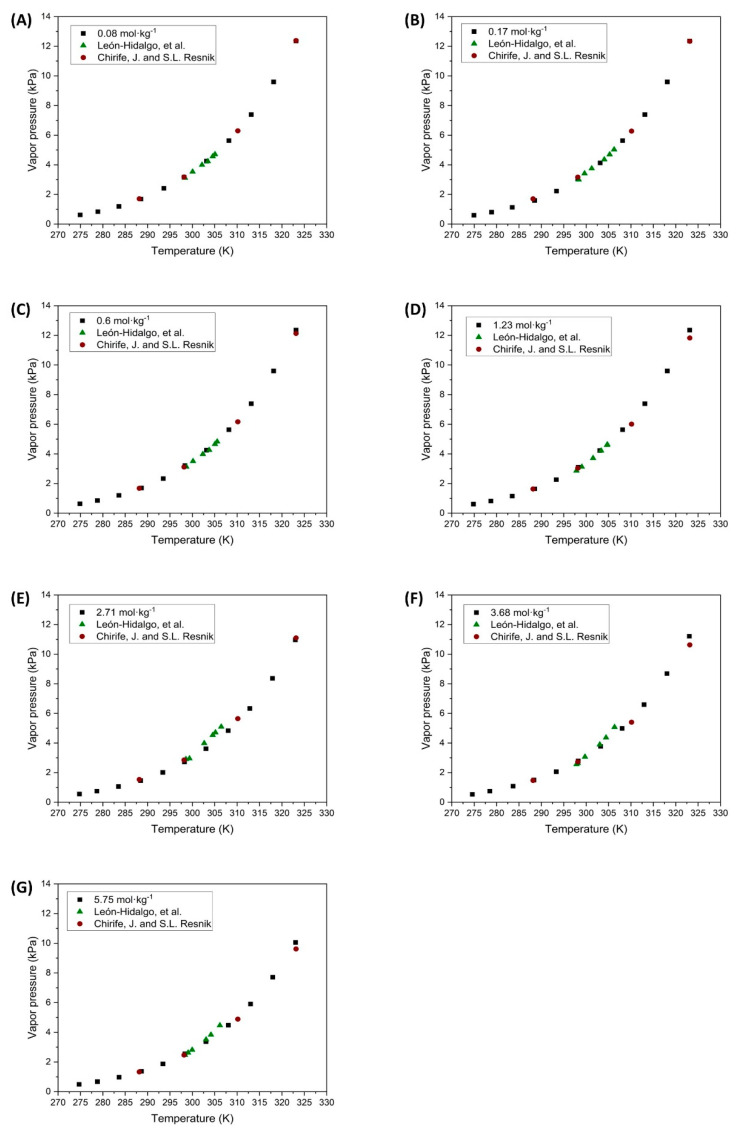
Vapor pressure of unsaturated NaCl solutions at different molalities. (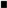 Experimental data; 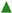 León-Hidalgo, et al. [41]; 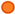 Chirife, J. and S.L. Resnik [46]).

The vapor pressures of NaCl solutions at different molalities have been experimentally determined and compared with values reported by other authors. The results for the unsaturated NaCl solutions are presented in Figure 4.

Based on the results obtained from determining the vapor pressures of unsaturated NaCl solutions, it can be concluded that these results align with those reported by other authors, as shown in Figure 4. The small deviations observed occur as the concentration increases from a molality of 2.71 mol·kg^−1^ to 5.75 mol·kg^−1^, when comparing the results with those reported by Leon-Hidalgo et al. [41]. The main differences found at intermediate temperatures may be attributed to measurement errors due to the instrumentation used by the various authors. At elevated temperatures, the primary discrepancy may arise from the calculation of *P_v,sat_* as previously mentioned. Despite these slight differences, the values obtained in the present investigation are considered to be accurate.

**Figure 5:**
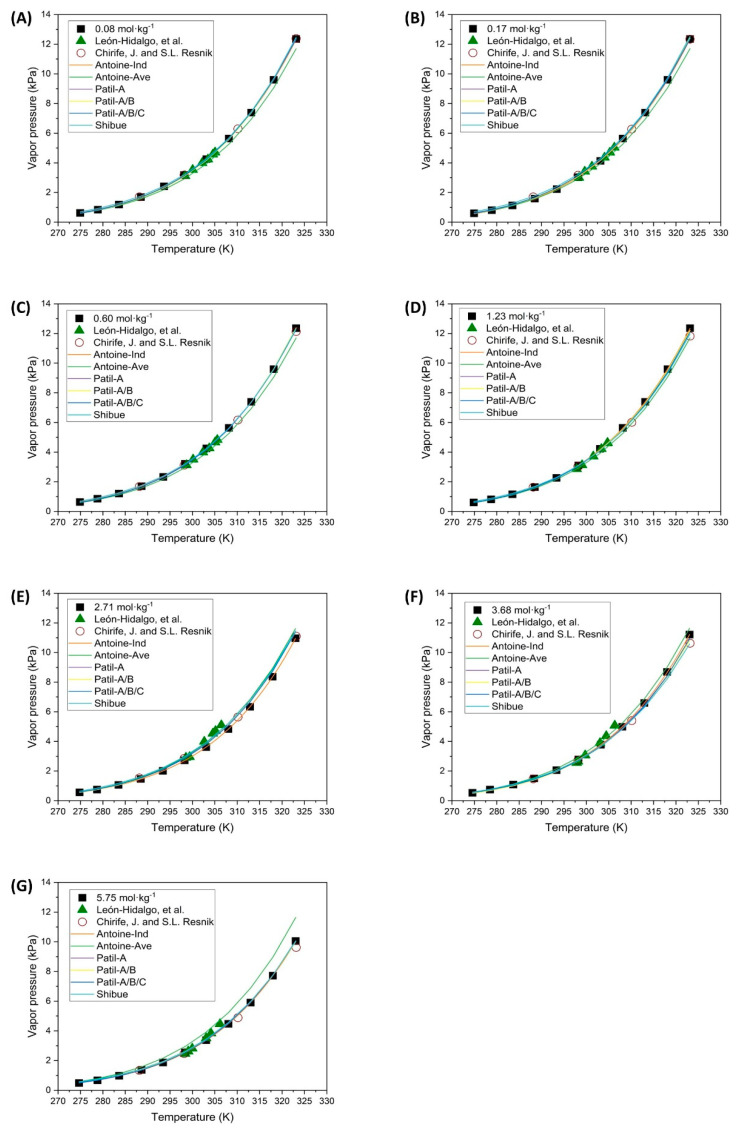
Vapor pressure of unsaturated NaCl solutions and different expressions for parameter fitting (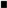 Experimental data; 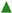 León-Hidalgo, et al. [41]; 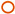 Chirife, J. and S.L. Resnik [46]; 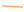 Individual fitting; 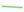 Average fitting; 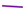 Patil-A; 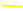 Patil-A/B; 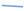 Patil-A/B/C; 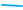 Shibue).

The parameters of the Antoine-type Equation (3) were fitted for each concentration separately as well as for all the concentrations together. This was done to investigate if there is any relationship between the parameters and the salt concentration that could explain the behavior of the vapor pressures without complicating the model. The values of the fitted parameters are shown in Table 4, and the representation of the fit is illustrated in Figure 5.

Observing the results obtained from the parameter fitting of Equation (3), Figure 5 and Table 4, it is clear that fitting each concentration separately provides a good representation of the experimental points. However, the fit of all concentrations together exhibits significant deviations. The fitted curve overestimates the experimental data for concentrations greater than 2.71 mol·kg^−1^. This discrepancy is also reflected in Table 4, where the residual error for the average fit is notably higher than that of each separate fit.

The fittings made for each concentration separately have resulted in distinct values for A, B, and C for each case. These fittings closely match the experimental points and exhibit a very low residual error, indicating that they can accurately represent the pattern of the experimental data. However, the drawback of this type of fit is that the Antoine equation does not explicitly include the concentration value, making the fit accurate only for each concentration individually. Therefore, a different model must be used to obtain an expression for determining vapor pressure at any concentration. Consequently, Patil’s model, as shown in Equation (4), is proposed for this purpose.

Patil’s expression, with the fitting parameters represented as cubic polynomials in terms of molality, has been fitted sequentially. This approach avoids complicating the fitting method and ensures the fitting values have acceptable confidence limits for the parameters. The first approach to fitting the parameters involved using Equation (4) but only considering parameter *A* as a function of molality. The results of the fitting parameters are shown in column *A(m)* of Table 5. The theoretical curves obtained from these parameters are illustrated in Figure 5, closely matching the experimental points for all the concentrations tested. Observing the values of the fitting parameters in Table 5, they are considered accurate as their confidence intervals are close to the estimated values, with the exception of parameters *B*_0_ and *C*_0_. Parameters *B_0_* and *C_0_* likely do not have acceptable confidence intervals because they do not significantly influence the vapor pressures in Equation (4). Comparing these results with those provided by Patil for the tested solutions, they are of the same order of magnitude and are thus deemed correct.

The second approach involves considering both parameters *A* and *B* of Equation (4) as functions of molality, with the estimated values recorded in column *A(m)* − *B(m)* in Table 5. In the third step, all three parameters (*A*, *B* and *C*) have been assumed to vary as functions of molality, and the results are shown in column *A(m)* − *B(m)* − *C(m)* of Table 5. The corresponding theoretical curves are depicted in Figure 5.

Based on the results obtained from the various parameterizations of Equation (4), it can be concluded that increasing the number of fitting parameters leads to poorer confidence intervals, despite minimal changes in residuals. This trend suggests that using *A*, *B* and *C* simultaneously as functions of molality is not justified, especially given the significant errors observed for parameters *B* and *C*. Upon reviewing Figure 5, it can be concluded that the simplest approach is the most appropriate and the parameters presented in column *A(m)* of Table 5 are the most suitable for determining vapor pressures as functions of temperature and concentration.

Finally, Equation (8) proposed by Shibue [30] was used as the last fitting expression, employing either 3 (*a_1_*, *a_2_*, *a_3_*) or 5 (*a_1_*, *a_2_*, *a_3_*, *b_1_*, *b_2_*) fitting parameters depending on whether the diluted or concentrated region was considered. The estimated parameters obtained with the equations corresponding to the diluted region are shown in Table 6 and the theoretical curves in Figure 5. These parameters present values with highly acceptable confidence limits. Moreover, this fitting expression yields a considerably lower error than other tested expressions, and notably, it serves effectively as a fitting model for diluted and concentrated regions. However, attempts to fit the parameters for the equations associated with the concentrated region were unsuccessful. Additionally, using Shibue’s expression with a quadratic difference in logarithmic vapor pressures also did not yield a successful fit. The fit of parameters in the dilute region is considered accurate, as they are similar to those obtained by Shibue, except for *a_3_*, which in the current investigation is an order of magnitude lower than that reported by Shibue.

In conclusion, the best fits are those with Equations (8)–(11) shown by Shibue. With just three fitting parameters, this approach perfectly explains the behavior of the vapor pressures of the solutions across the entire range of concentrations and temperatures tested. Next, Patil’s expression modified using only the combination of Equations (4) and (5), where only the fitting parameter *A* is assumed as a function of molality, is also considered effective. With only 6 fitting parameters, this expression results in a better fit with more restrictive confidence intervals. Finally, while Antoine’s expression (3) is deemed correct, its limitation is that the fitted values are only valid for the specific concentrations of the solution used, as it does not account for the effect of concentration on the fitting parameters.

#### 3.2.2. CuSO_4_·5H_2_O Solutions

**Figure 6:**
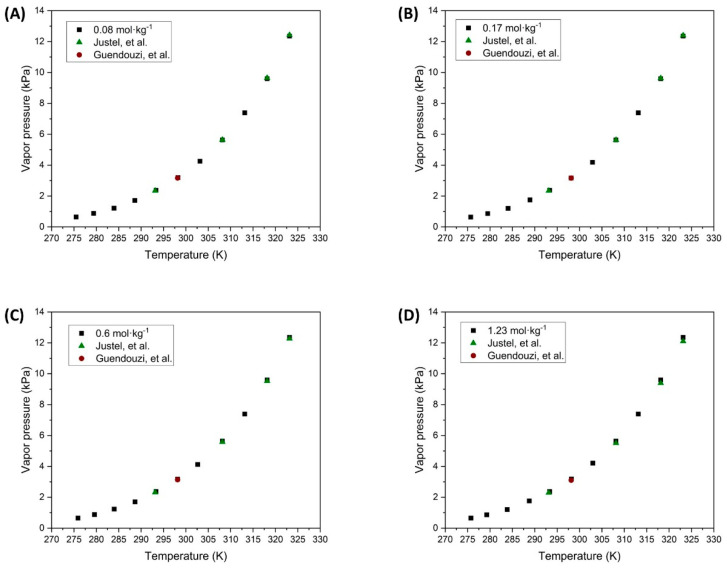
Vapor pressure of unsaturated CuSO_4_·5H_2_O solutions at different molalities. (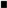 Experimental data; 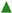 Justel, et al. [47]; 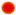 Guendouzi, et al. [48]).

The results obtained for the unsaturated CuSO_4_·5H_2_O solutions were carried out up to a molal concentration of 1.23 mol·kg^−1^, because its solubility is highly dependent on temperature. At a molal concentration of 2.71 mol·kg^−1^ the solution is in a saturated state, so the tests were conducted for the first four molal concentrations studied. The results of these tests for the range of working temperatures are depicted in Figure 6.

Based on the results obtained, it can be seen that the experimental data from this investigation closely align with literature values within the working temperature range. However, a slight deviation is observed at temperatures around 50 °C, corresponding to the experimental values reported by Justel et al. [47]. As previously mentioned, this drift in the experimental measurement may be due to the vapor pressure expression of pure water used. Conversely, the data at 25 °C and lower temperatures agree with the experimental points reported by Guendouzi et al. [48].

In the tests carried out in the present investigation, it was observed that the relative humidity of the air, measured with the hygrometer, showed 100% saturation when the equilibrium temperature of 20 °C was reached, regardless of the solution concentration. This observation can be directly related to the stability of the salt with hydrated pairs, as reported by Glasser [49]. This may be because as the salt has a larger hydration sphere and its solubility increases with temperature, it is easier for these water molecules to dissociate from the main molecule and evaporate. Consequently, the CuSO_4_ salt forms smaller hydrated pairs resulting in the environment reaching 100% relative humidity.

**Figure 7:**
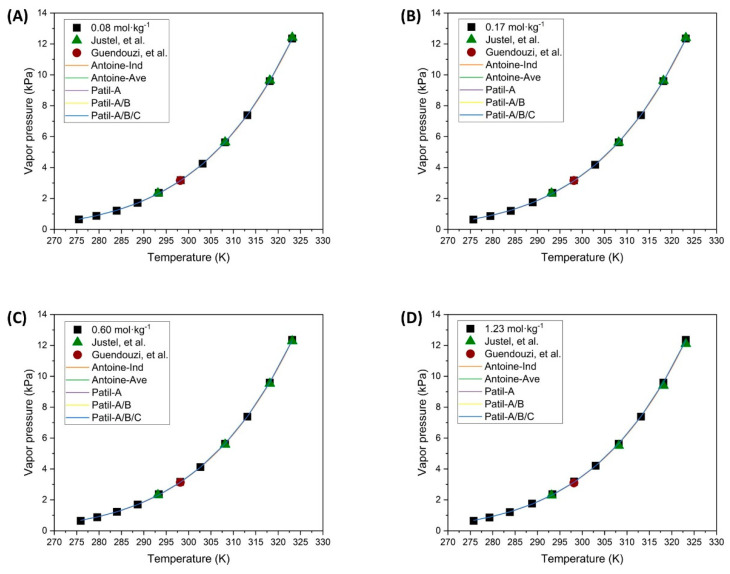
Vapor pressure of unsaturated CuSO_4_·5H_2_O solutions and different expressions for parameter fitting (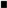 Experimental data; 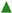 Justel, et al. [47]; 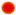 Guendouzi, et al. [48]; 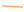 Individual fitting; 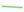 Average fitting; 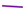 Patil-A; 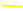 Patil-A/B; 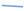 Patil-A/B/C).

The parameter fitting for the vapor pressure expressions at different concentrations has been performed similarly to the NaCl solutions, starting with the Antoine Equation (3) for each concentration separately and for all concentrations simultaneously. The results of this parameter fitting are presented in Table 7, and the corresponding theoretical curves are shown in Figure 7.

As seen in the results obtained, the fitting parameters for the different concentrations are not drastically affected, and even the estimated parameters for all concentrations fall within the range of values of the other settings. In contrast, observing the fitting error and the theoretical curves, it can be concluded that the fit is accurate. This simple expression effectively describes all concentrations of this solution, even though the concentration is not explicitly included in the fitting expression.

The Patil model has been used to validate a more complex expression that incorporates the molality of the solutions tested in this research. The fitting procedure was conducted similarly to that for the NaCl solution, beginning with a simpler expression where only parameter *A* depends on the concentration and subsequently applying equations where all parameters depend on the concentration. The results of the fitted parameters are presented in the corresponding column of Table 8. The theoretical curves obtained are illustrated in Figure 7.

According to the parameter values obtained, the same trend observed with the NaCl solution can be seen: as the number of fitting parameters increases their corresponding confidence intervals also expand, but the residuals are not improved. Additionally, it is notable that even with the simplest expression it is possible to accurately describe the vapor pressures that would be obtained in any case.

Observing the results obtained from the fitting expressions and the theoretical curves in Figure 7, it was decided not to use the fitting of Equation (8) proposed by Shibue, since the previous estimations have already produced theoretical curves that faithfully represent the experimental data of the present investigation, as seen in Figure 7. The theoretical curves from each fitting are practically superimposed, with minimal differences. Therefore, it can be concluded that for the different concentrations of the CuSO_4_·5H_2_O solution, the Antoine-type Equation (3) for the average values shown in Table 7 is sufficient to predict the vapor pressures of this solution. Conversely, the fittings made using Equation (4) are not considered adequate due to the large errors in the confidence intervals, despite the curves overlapping the experimental points.

### 3.3. Evaporation of Saturated Salts on Different Free Evaporating Surfaces

**Figure 8:**
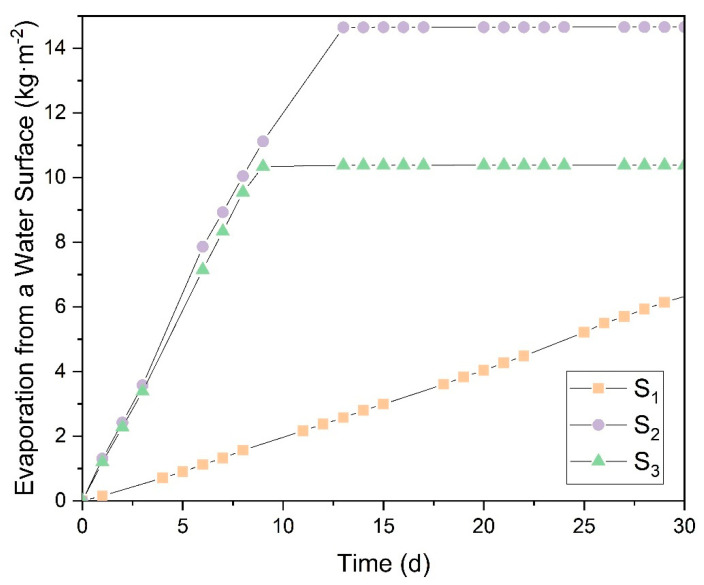
Evaporation of water in saturated NaCl solution for three evaporating surfaces. (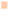 S_1_ (141.2 mm^2^); 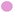 S_2_ (1683.7 mm^2^); 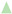 S_3_ (3452.4 mm^2^)).

Figure 8 presents the results for the natural evaporation of a saturated NaCl solution across different evaporation surfaces: *S_1_* (141.2 mm^2^), *S_2_* (1683.7 mm^2^), and *S_3_* (3452.4 mm^2^). The data show that surface *S_1_* exhibits distinct behavior in evaporation per unit area and requires a longer time to fully evaporate the water compared to *S_2_* and *S_3_*, with *S_1_* requiring approximately 180 days to complete the process. For surfaces *S_2_* and *S_3_*, the difference in evaporation per unit area becomes noticeable only after the complete evaporation of the water, as indicated by the plateau in the curves. Initially, the slopes for *S_2_* and *S_3_* are identical, indicating that the evaporation surface area does not significantly impact the evaporation rate. Instead, increasing the surface area results in a higher total mass of water evaporated, but does not alter the evaporation per unit area.

**Figure 9:**
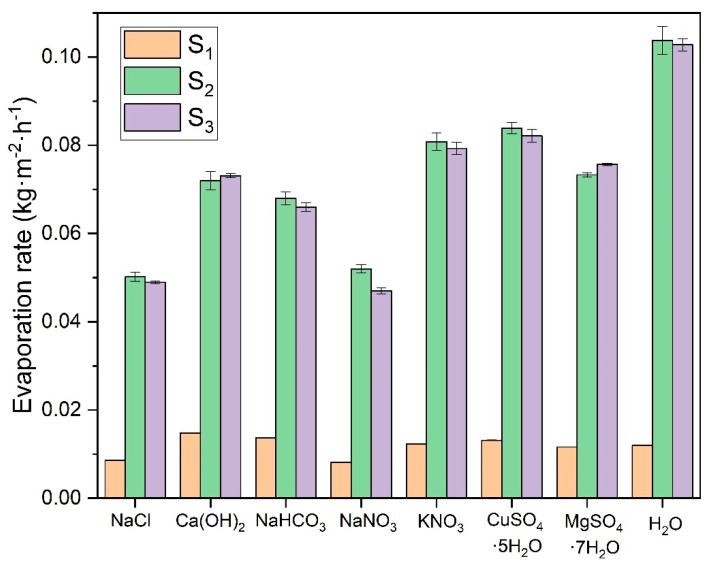
Evaporation rates of saturated solutions for three evaporating surfaces. (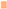 S_1_ (141.2 mm^2^); 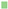 S_2_ (1683.7 mm^2^); 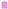 S_3_ (3452.4 mm^2^)).

The other salts studied for natural evaporation exhibited similar behavior to that shown in Figure 8, so this figure is presented primarily to illustrate the experimental test conducted. To compare the results of the different saturated salt solutions, the slopes of the evaporation curves during the initial linear evaporation phase, corresponding to the first days of evaporation, were analyzed. These results are displayed in Figure 9, which compares the evaporation rates of all solutions tested in this research, including distilled water.

The results indicate no significant difference in the evaporation rates between surfaces *S_2_* and *S_3_*, as their values are nearly identical across all study cases, with any minor differences likely attributable to measurement errors. However, a notable difference is observed between these larger surfaces (*S_2_* and *S_3_*) and surface *S_1_*, with a reduction in the evaporation rate ranging from 82.7% to 92.2%. In contrast, despite having twice the evaporation area, *S_2_* and *S_3_* exhibit similar evaporation rates, suggesting that there may be a critical surface area at which the evaporation rate stabilizes regardless of further increases in surface area.

Assouline et al. [50] experimented to determine water evaporation in two tanks. A black polypropylene plate covered the surface of one of the tanks with evenly distributed square perforations. This design allowed the use of different evaporation areas in the experiment. Assouline et al. demonstrated that with a very small evaporation surface, the evaporation rate deviates from the ideal trend. As the surface area increases, it approaches the maximum evaporation rate. Therefore, it can be concluded that there is a limiting surface area, beyond which further increases do not affect the evaporation rate. This effect may explain the behavior observed for surface *S_1_*, which deviates from the ideal evaporation trend. In contrast, surfaces *S_2_* and *S_3_* exhibit the maximum possible evaporation rate.

The different salts’ evaporation rates depend mainly on their vapor pressure in equilibrium with the air temperature. In Section 3.1, the vapor pressures of these saturated solutions are determined. Comparing the vapor pressure results with the evaporation rate experiments confirms a good correlation between both variables. The CuSO_4_·5H_2_O and MgSO_4_·7H_2_O solutions exhibit the highest evaporation rates due to their hydration equilibrium state, which allows them to eliminate excess water molecules from the solution at a higher rate regardless of their vapor pressure. It should be noted that when calculating the evaporation rates in the linear evaporation interval, these solutions have the highest values. However, as they reach equilibrium with the ambient humidity, they do not completely evaporate the water from their structure and can even gain mass if the environmental humidity increases. This effect is evident when these solutions have almost no apparent liquid left in the vessel and salt deposits become noticeable. In contrast, other solutions with high vapor pressures dried out completely during the experiment, maintaining a constant evaporation rate throughout the trial. The evaporation rate of these hydrated salts drops drastically once they reach equilibrium with the environment. Genceli, F. E. et al. [51] investigated MgSO_4_·nH_2_O salt in different hydration states, determining that at room temperature and depending on the relative humidity it can overhydrate up to 11 times. This demonstrates that these hydrated salts can retain water in their crystalline structure.

KNO_3_ shows a slightly higher evaporation rate than NaHCO_3_ and Ca(OH)_2_, while lower evaporation rates characterize NaCl and NaNO_3_. Comparing all these results with those of distilled water, it can be seen that this shows the highest evaporation rate since it evaporates on a free film, and its rate is influenced primarily by environmental conditions such as temperature and relative humidity.

This experimental test is used to determine the evaporation rates of salts in the saturated state, representing the worst possible concentration conditions for the evaporation of the water present in the solutions. Gilron et al. [25] determined an evaporation rate for the volume reduction of waste from brackish water, where they obtained values of evaporation rates from the free surface of water of 0.0796 kg·m^−2^·h^−1^. This value approximates the results obtained for the solutions in the present investigation. More recent studies by Gilron et al. [52], investigating evaporation rates for brines concentrated to a state of ZLD or almost no liquid, found an approximate evaporation rate of 0.0459 kg·m^−2^·h^−1^, for the water-free film. This is similar to the values obtained in the present study for the saturated NaCl solution, with an average evaporation rate of 0.0496 kg·m^−2^·h^−1^.

### 3.4. Evaporation of NaCl and CuSO_4_·5H_2_O Saturated Solutions in Fabrics

**Figure 10:**
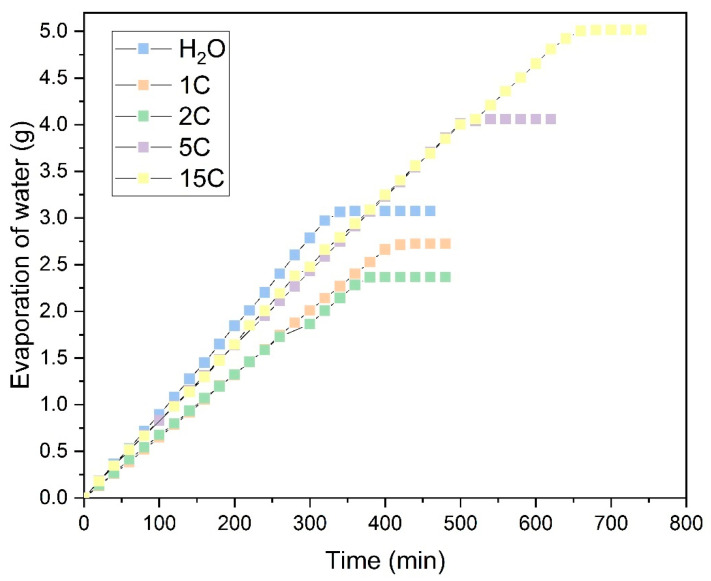
Evaporation of water from the saturated NaCl solution in the Ara sample. (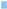 Distilled water; Evaporation cycles 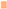 1C; 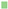 2C; 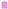 5C; 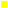 15C).

As previously mentioned, evaporation studies on natural and synthetic fabrics have been carried out using saturated NaCl and CuSO_4_·5H_2_O solutions on samples with a 50 mm × 75 mm surface area and an effective evaporation area of 50 mm × 60 mm. These two salts were chosen for determining evaporation rates due to their distinct properties: NaCl, with the lowest vapor pressure, and CuSO_4_·5H_2_O, which is a structurally larger salt in its hydrated state. Figure 10 shows the evaporation process for the Ara sample in NaCl solution across different evaporation cycles.

**Figure 11:**
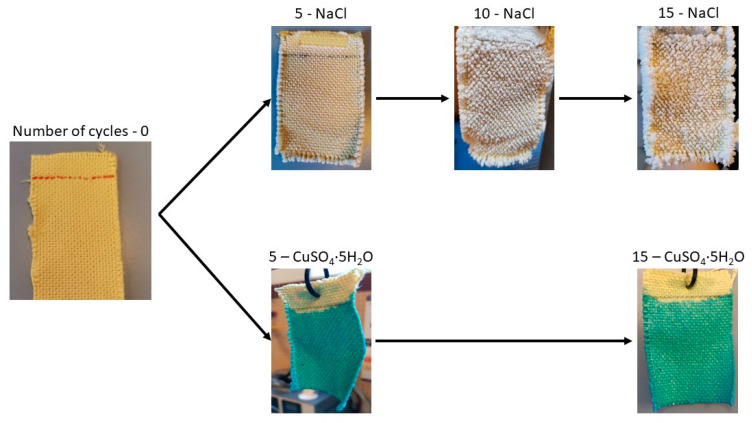
Precipitation of NaCl and CuSO_4_·5H_2_O salts on Ara fabric along 15 cycles of evaporation.

In these tests, only the evaporation of water was accounted for, with the salts precipitated on the fabric as the water evaporated being discounted. The graphs compare the evaporation per cycle of each saturated solution with that measured in distilled water. In the NaCl solution, shown in Figure 10, it can be observed that during the first few operation cycles (1C and 2C), the water absorbed by the fabric and measured in evaporation is similar to that of distilled water. This suggests that salt precipitation does not initially affect evaporation. As the number of evaporation cycles increases, NaCl precipitates within the fabric structure. This effect is illustrated in Figure 11, showing NaCl crystals growing perpendicularly to the fabric surface, particularly at the edges of the sample. This salt precipitation causes an increase in water absorption due to the combined effects of the fabric’s inherent absorption and the hydration of the precipitated crystals. This results in increased liquid absorption and, consequently, higher total water evaporation, as seen in Figure 10.

**Figure 12:**
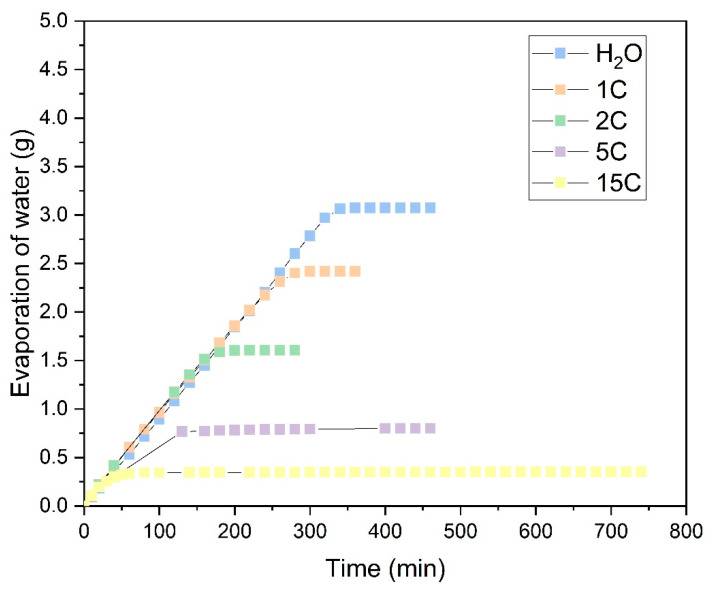
Evaporation of water from the saturated CuSO_4_·5H_2_O solution in the sample Ara. (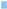 Distilled water; Evaporation cycles 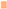 1C; 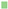 2C; 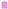 5C; 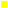 15C).

Figure 12 shows the tests conducted with CuSO_4_·5H_2_O salt and highlights a completely different behavior compared to the NaCl salt observed previously. As can be seen, compared to evaporation with distilled water, the absorption capacity reduces from the first cycle onwards and continues to decline with subsequent evaporation cycles. This reduction is likely due to the pentahydrated nature of the salt and to the formation of CuSO_4_ crystals. As shown in Figure 11, these crystals do not grow in the same manner as NaCl crystals, with little difference between the 5C and 15C cycles. The precipitation of CuSO_4_ forms a dense, solid layer that prevents the solution from penetrating into the fabric structure during each operation cycle and inhibits the fabric’s ability to retain water. From cycle 5C onwards, the mass of salt precipitated on the fabric remains consistent at approximately 0.1 g, indicating that the saturated CuSO_4_·5H_2_O solution is retained only on the CuSO_4_·5H_2_O crystals already precipitated in the previous cycle.

Only the graphs showing the water evaporation over time for the Ara sample are presented, as its trend mirrors that of the other fabrics tested. Next, the evaporation rates for all samples are analyzed during each work cycle, focusing on the range where the evaporation rate is at its maximum. In this zone, a linear evaporation trend with a constant slope has been observed, as indicated in the previous figures, until complete evaporation is achieved.

**Figure 13:**
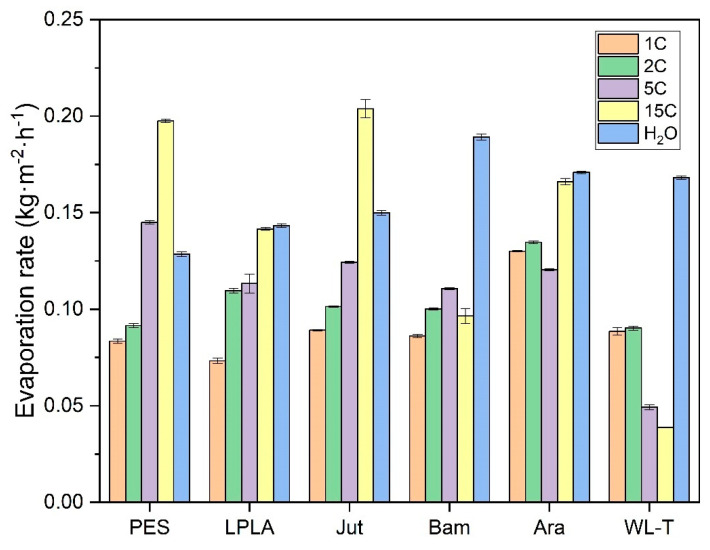
Evaporation rate in saturated NaCl solution for the different fabrics in the zone of maximum evaporation. (Evaporation cycles 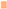 1C; 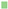 2C; 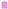 5C; 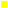 15C; 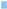 Distilled water).

The results for the saturated NaCl solution are shown in Figure 13. The general trend observed is consistent with that of the Ara sample. Both natural and synthetic fabrics exhibit an increase in evaporation rate as the number of cycles increases, with some fabrics even surpassing the evaporation rate of distilled water (PES and Jut). Two natural fabrics, Bam and WL-T, display different behaviors. The Bam sample, with a very low fabric weight of 0.2193 g, shows minimal salt retention, making the effect of additional hydration as evaporation cycles increase less noticeable. In contrast, the WL-T fabric exhibits a decreasing trend in evaporation rate as the cycles progress. This reduction may be attributed to the interaction between the saturated NaCl solution and the palm fibers, which could occlude voids between the fibers and increasingly restrict water evaporation from the fabric to the environment.

Observing the results obtained in these initial experiments with the NaCl saturated solution, the most promising fabrics for long-term evaporation cycles are PES, LPLA, Jut, and Ara. At around 15 evaporation cycles, these fabrics exhibit an increased evaporation rate, often surpassing that of distilled water due to the hydration of NaCl crystals deposited on their structure. Conversely, the Bam fabric stands out for applications requiring low salt retention. Despite its stable evaporation rate throughout the cycles, its salt retention is significantly lower than that of the other tested fabrics.

In the present investigation, it has been observed that evaporation rates for distilled water range between 0.1285 to 0.1892 kg·m^−2^·h^−1^ under ambient laboratory conditions, with a temperature range of 20 ± 2 °C, relative humidity of 40 ± 5 % and zero air velocity. Authors such as Shokri Kuehni et al. [53] state that environmental conditions of temperature and relative humidity significantly affect the evaporation rate from porous media. Shokri Kuehni et al. conducted tests at a constant temperature with increasing relative humidity and observed a decrease in the evaporation rate. Conversely, evaporation is favored at constant relative humidity with an increase in temperature.

Liu et al. [54] determined evaporation rates for polyester fabrics under conditions simulating human skin, at a temperature of 36 °C, relative humidity of 40%, and a wind speed of 0.3 m·s^−1^. Because these conditions are more favorable than those tested in the present study, evaporation rates of 0.2544 to 0.3078 kg·m^−2^·h^−1^ were obtained, approximately twice the values reported in this paper. This difference is explained by the temperature and wind speed variations at which the experimental tests were conducted.

Some authors who have worked under similar conditions include Fourt et al. [55], who studied hand-made fabrics at a temperature of 21 °C and a relative humidity of 65%, obtaining evaporation rates of 0.045 to 0.072 kg·m^−2^·h^−1^. On the other hand, Gurudatt et al. [56] carried out tests in ambient conditions of 25 °C and relative humidity of 64%, obtaining evaporation rates of 0.1062 to 0.1158 kg·m^−2^·h^−1^. In the present work, slightly higher evaporation rates than those shown by the other authors have been measured at a lower relative humidity of 40%.

Alternatively, the studies of Wu et al. [57], who investigated evaporation in saline media with surface-treated cotton fabrics, are worth mentioning. They worked at temperatures of 25 °C and relative humidities of 35 to 55%, obtaining evaporation rates of 0.166 kg·m^−2^·h^−1^. These values are more similar to those obtained in the present research, with a difference of 12%, although they are different solutions.

In the previous cases analyzed, only the environmental factors of temperature and relative humidity, which critically affect the evaporation rate, have been compared. Other factors influencing the evaporation rate may include the weight of the fabrics, as indicated by Chau et al. [58]. These authors suggest that as the grammage of a fabric increases, the evaporation rate is reduced. Crow and Osczevski [59] showed that fabrics can absorb more liquids if their thickness is increased and that the drying time of the samples depends on the grammage and thickness, as these factors directly influence the fabric’s ability to retain more liquids in their structure.

Figure 13 also shows that during the evaporation cycles for the saturated solution of NaCl, some of the fabrics exhibit an evaporation rate higher than that of water. This effect sounds counterintuitive, as the vapor pressure of this salt is quite low and, therefore, should not exceed that of distilled water. Shokri-Kuehni et al. [60] demonstrated in their experiments that the mass loss over days in a porous medium for a concentrated NaCl solution was lower than when it was more diluted. In their research, Norouzi Rad and Shokri [61] observed that adding NaCl in a porous medium up to a concentration of approximately 1.5 mol·kg^−1^ decreased the evaporation rate. However, above this point, increasing the concentration caused an increase in the evaporation rate. This effect of increasing the evaporation rate in a porous medium was also demonstrated by Sghaier and Prat [62], who observed that the evaporation rate for a saturated NaCl solution was higher than that of pure water in the first hours of evaporation, until the crystallization phase of the salt crystals was reached.

**Figure 14:**
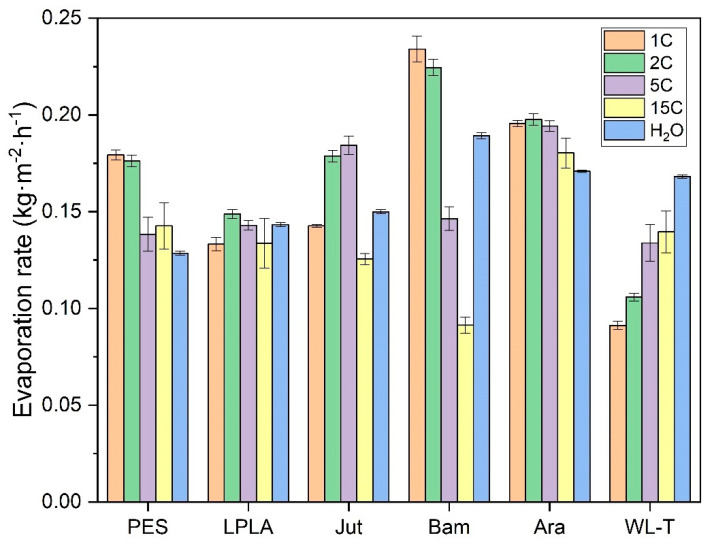
Evaporation rate in saturated CuSO_4_·5H_2_O solution for the different fabrics in the zone of maximum evaporation. (Evaporation cycles 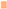 1C; 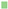 2C; 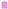 5C; 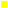 15C; 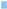 Distilled water).

Subsequently, the same type of fabrics were used for evaporation tests in a saturated CuSO_4_·5H_2_O solution. The values of the evaporation rates determined in the zone of maximum evaporation can be seen in Figure 14. Although the time for complete evaporation after five cycles of operation increases dramatically compared to the saturated NaCl solution tests, the evaporation rate shows higher average values than the NaCl solution. This effect is mainly due to the vapor pressure of each solution at the temperature at which each test was performed. In this case, the vapor pressure associated with CuSO_4_·5H_2_O is higher than that of NaCl at the same temperature. This effect causes the evaporation rate of the water to be faster until it reaches the crystallization state of the salts, where the time for complete evaporation of the CuSO_4_·5H_2_O salt is higher than that of NaCl at the same temperature.

As seen in the results obtained, the maximum evaporation rate obtained in the first cycles of the tests carried out for the CuSO_4_·5H_2_O salt is not drastically affected, like the NaCl salt, showing a reduction as the evaporation cycles increase. The Bam sample stands out due to its small weight, causing the CuSO_4_·5H_2_O salt to crystallize within the fabric structure and completely block the gaps between the fibers, which reduces its evaporation rate. This is because it absorbs little solution and quickly moves to the crystallization phase. On the other hand, the WL-T fabric slightly increases its evaporation rate because the fibers on its surface are not completely blocked and the surface roughness is able to retain some more solution in its structure without reaching the crystallization phase.

Although the drying times for the fabrics in the presence of the saturated CuSO_4_·5H_2_O solution are longer, we focused on the initial evaporation phase, where the salt has not yet precipitated, and the evaporation rates remain stable initially, decreasing in the later cycles. This is due to the high vapor pressure of CuSO_4_·5H_2_O, which is similar to that of pure water at temperatures of 20–25 °C until the nucleation phase. De Castelnuovo et al. [63] and Saig et al. [64] explain the dehydration mechanisms of CuSO_4_·5H_2_O in equilibrium with CuSO_4_·3H_2_O + 2H_2_O, highlighting that at high temperatures, excess water converts to gas and reabsorbs to maintain equilibrium. This could explain the slow drying observed in experiments with high evaporation cycles. Additionally, Donkers et al. [65] demonstrated through thermal decomposition by thermogravimetric analysis (TGA) that CuSO_4_·5H_2_O is highly stable with its five water molecules, indicating that excess water can be eliminated while decomposition does not occurs, achieving high evaporation rates.

It was decided to compare the evaporation rates between cycles and saturated salts used in the different fabrics in the first cycle (1C), the last cycle (15C), and in comparison with distilled water (Table 9).

As can be observed, the tendency in the NaCl cycles is to increase the evaporation rate due to the increase in water absorption, even surpassing the evaporation rate of the CuSO_4_·5H_2_O solution despite having a lower vapor pressure than this solution. In contrast, the CuSO_4_·5H_2_O solution shows a slight reduction in its evaporation rate over the fifteen cycles calculated in the zone of the highest evaporation rate.

In view of the results obtained from the evaporation tests using natural and synthetic fabrics, a summary of the conditions that most affect the evaporation rate has been compiled. These factors are listed in Table 10 in order of importance, considering that the evaporation tests were conducted with negligible or no air velocity.

When treating wastewater or saline water with a significant presence of salts, the factors mentioned in Table 10 should be considered to select the fabric that best performs the process under study.

## 4. Conclusions

The determination of vapor pressures of saturated solutions has shown similar trends and results to those obtained by other authors mentioned in this research, with slight deviations at elevated temperatures in the range of 35 to 50 °C. These deviations are probably due to the fitting expression for the vapor pressure of pure water. However, there is no available information from other researchers on the Ca(OH)_2_ and NaHCO_3_ solutions. The trend shown by these two solutions is similar, as both have an equilibrium relative humidity higher than 95% from a temperature of 15 °C, indicating a vapor pressure closely that of pure water.

On the other hand, the determination of vapor pressures for unsaturated NaCl solutions has shown the expected trend: as the concentration of the solution increases, the vapor pressure decreases, making evaporation more difficult in open environments. For CuSO_4_·5H_2_O solutions at concentrations below saturation, all tested molal concentrations have shown, starting at a temperature of 20 °C, a relative humidity of 100%.

Parameter fitting conducted for unsaturated NaCl and CuSO_4_·5H_2_O solutions at various concentrations has shown that the Antoine-type expression needs to be fitted for each solution separately. However, for CuSO_4_·5H_2_O, an average fit can accurately represent the theoretical results. On the other hand, Patil’s expression is noteworthy because it can accurately describe the results with a simpler form across all concentrations. Finally, Shibue’s expression is particularly useful for NaCl concentrations. In the dilute region, using three fitting parameters, the behavior was effectively captured across the entire range of concentrations studied.

The evaporation of saturated solutions of the salts used in this research from free water surfaces has shown the expected effect: they all reduce vapor pressure, resulting in slower evaporation rates compared to distilled water. NaCl exhibited the slowest evaporation rate among these salts due to its lowest vapor pressure. Conversely, CuSO_4_·5H_2_O and MgSO_4_·7H_2_O showed higher evaporation rates when considering the maximum evaporation rate. However, both salts do not completely evaporate the water occluded in their structure once they reach equilibrium with the environment. This drastically reduces their evaporation rate and can even increase their mass if the environmental humidity is high. Additionally, the effect of the evaporation surface indicates that there is a critical surface area beyond which it does not influence the evaporation rate, as observed in the differences between surface S_1_ and surfaces S_2_ and S_3_ in all cases.

Finally, studies conducted to determine the evaporation of water on natural and synthetic fabrics using saturated NaCl and CuSO_4_·5H_2_O solutions have shown different behavior than on free water surfaces. On the one hand, for the saturated NaCl solution on the fabrics, it was observed that as the evaporation cycles increase and more salt deposits on the fabrics, the more water evaporates from the fabrics, the greater the evaporation rate and the higher the salt deposited on the fabrics. In some cases, such as the PES and Jut samples, the evaporation rate in cycle 15C exceeds that of distilled water, despite NaCl having a lower vapor pressure than pure water at the working temperature. Conversely, the CuSO_4_·5H_2_O salt exhibited an opposite effect. As the evaporation cycles increase, the absorption capacity of the fabric decreases because the salt precipitates on the fabric structure, blocking its surface and internal structure. This obstruction makes it difficult for the liquid solution to interact with the fabric, causing the solution to remain in contact with the salt deposited from previous cycles. The maximum evaporation rate determined for CuSO_4_·5H_2_O is sometimes higher than that of water due to its overhydration effect, having more than five water molecules in its structure. When evaporation begins, this excess water starts to precipitate on the fabric, reaching equilibrium and drastically reducing the evaporation rate, resulting in very long periods for complete evaporation.

The findings presented in this study establish a foundation for the development of mathematical models of evaporation, particularly those involving natural and synthetic fabrics, as well as the evaporation of water masses in free surface films. This research has systematically examined the vapor pressures of both saturated solutions and solutions at varying concentrations, enabling the precise calibration of vapor pressure models across a broad temperature spectrum. These parameters are crucial for advancing our understanding and will serve as a basis for the development of both simple and complex evaporation models that utilize vapor pressure as the driving gradient.

In addition, the investigation into evaporation using natural and synthetic fabrics has provided valuable insights into the behavior of different materials under extreme conditions in the context of saturated solution treatment. These findings will facilitate the selection of fabrics with optimal performance characteristics for wastewater treatment, with potential implications for industrial-scale applications.

## Figures and Tables

**Table 1:** Evaporation surfaces used in the natural evaporation of saturated solutions.

Type of Vessel Used	Diameter (mm)	Evaporation Surface (mm^2^)
Small (S_1_)	13.4 ± 0.3	141.2 ± 5.7
Medium (S_2_)	46.3 ± 0.3	1683.7 ± 25.2
Large (S_3_)	66.3 ± 0.3	3452.4 ± 36.1

**Table 2:** Fitted values for the P_c_ expression determined by Shibue [30].

Parameter	Value
*q_5_*	9.00404 × 10^2^
*q_6_*	−2.92542 × 10^4^
*q_7_*	1.39806 × 10^6^
*q_8_*	−2.80756 × 10^7^
*q_9_*	2.41637 × 10^8^
*q_10_*	−7.18726 × 10^8^

**Table 3:** Values for the fitted parameters of Antoine’s equation.

Saturated salt	*A*	*B*	*C*	Residual
Ca(OH)_2_	14.5 ± 8.40 × 10^−1^	2.91 × 10^3^ ± 3.80 × 10^2^	−81.0 ± 14.8	2.00 × 10^−3^
NaHCO_3_	15.3 ± 1.66	3.30 × 10^3^ ± 8.07 × 10^2^	−65.5 ± 29.7	5.00 × 10^−3^
NaCl	16.2 ± 9.40 × 10^−1^	3.84 × 10^3^ ± 4.91 × 10^2^	−46.9 ± 16.7	1.00 × 10^−3^
MgSO_4_·7H_2_O	20.1 ± 5.02	5.80 × 10^3^ ± 3.16 × 10^3^	6.53 ± 85.9	1.50 × 10^−3^
NaNO_3_	18.6 ± 1.76	5.21 × 10^3^ ± 1.07 × 10^3^	−3.43 ± 31.4	1.00 × 10^−3^
KNO_3_	18.3 ± 2.17	4.90 × 10^3^ ± 1.28 × 10^3^	−13.1 ± 38.5	4.00 × 10^−3^
CuSO_4_·5H_2_O	14.5 ± 9.30 × 10^−1^	2.86 × 10^3^ ± 4.17 × 10^2^	−84.9 ± 16.3	2.00 × 10^−3^

**Table 4:** The fitted parameters of Antoine’s Equation (3) for each molality separately and also for all molalities together with the NaCl solution.

	Parameters			
Molality (mol·kg^−1^)	*A*	*B*	*C*	Residual
0.08	13.9 ± 1.22	2.67 × 10^3^ ± 5.33 × 10^2^	−89.4 ± 21.9	5.00 × 10^−3^
0.17	12.9 ± 1.24	2.19 × 10^3^ ± 4.85 × 10^2^	−1.13 × 10^2^± 21.7	7.00 × 10^−3^
0.60	14.1 ± 8.70 × 10^−1^	2.76 × 10^3^ ± 3.86 × 10^2^	−86.2 ± 15.6	2.00 × 10^−3^
1.23	13.2 ± 9.60 × 10^−1^	2.32 × 10^3^ ± 3.90 × 10^2^	−1.06 × 10^2^ ± 17.1	4.00 × 10^−3^
2.71	19.2 ± 1.17	5.43 × 10^3^ ± 7.23 × 10^2^	−3.10 × 10^−1^ ± 20.5	1.00 × 10^−3^
3.68	15.9 ± 1.43	3.60 × 10^3^ ± 7.21 × 10^2^	−56.9 ± 25.2	3.00 × 10^−3^
5.75	16.0 ± 1.27	3.70 × 10^3^ ± 6.48 × 10^2^	−52.9 ± 22.4	2.00 × 10^−3^
All Exp. data	15.0 ± 8.69	3.13 × 10^3^ ± 4.09 × 10^3^	−73.1 ± 1.54 × 10^2^	12.9

**Table 5:** Parameter fitting to all experimental NaCl vapor pressure data for Equation (4).

Parameters	*A(m)*	*A(m)* − *B(m)*	*A(m)* − *B(m)* − *C(m)*
*A_0_*	5.63 ± 1.96	5.35 ± 2.08	4.48 ± 4.21
*A_1_*	−2.30 × 10^−3^ ± 1.10 × 10^−2^	−1.41 × 10^−1^ ± 4.34 × 10^−1^	4.00 × 10^−1^ ± 9.19
*A_2_*	−5.80 × 10^−3^ ± 5.00 × 10^−3^	7.40 × 10^−2^ ± 1.98 × 10^−1^	3.50 × 10^−1^ ± 3.84
*A_3_*	6.00 × 10^−4^ ± 1.00 × 10^−3^	−1 × 10^−2^ ± 2.30 × 10^−2^	−6.00 × 10^−2^ ± 5.20 × 10^−1^
*B_0_*	−6.72 × 10^2^ ± 1.21 × 10^3^	−4.83 × 10^2^ ± 1.28 × 10^3^	24.8 ± 2.55 × 10^3^
*B_1_*		4.66 × 10^1^ ± 1.37 × 10^2^	−2.54 × 10^2^ ± 6.17 × 10^3^
*B_2_*		−26.5 ± 62.6	−2.11 × 10^2^ ± 2.81 × 10^3^
*B_3_*		3.48 ± 7.19	37.3 ± 2.48 × 10^2^
*C_0_*	−2.57 × 10^5^ ± 1.87 × 10^5^	−2.88 × 10^5^ ± 1.97 × 10^5^	−3.62 × 10^5^ ± 4.02 × 10^5^
*C_1_*			3.99 × 10^4^ ± 8.73 × 10^5^
*C_2_*			3.10 × 10^4^ ± 3.72 × 10^5^
*C_3_*			−5.53 × 10^3^ ± 4.97 × 10^4^
			
Residuals	1.08	1.14	1.04

**Table 6:** Fitting parameters for all experimental values of NaCl vapor pressure for Equation (8).

Parameter	Value	Residual
*a_1_*	2.66 × 10^−1^ ± 3.60 × 10^−2^	2.63 × 10^−6^
*a_2_*	−2.25 ± 4.62 × 10^−1^	
*a_3_*	−43.5 ± 14.2	

**Table 7:** The fitted parameters of Antoine’s Equation (3) for each molality separately and also for all molalities together with the CuSO_4_·5H_2_O solution.

Molality (mol·kg^−1^)	Parameters			
	*A*	*B*	*C*	Residual
0.08	13.9 ± 1.24	2.66 × 10^3^ ± 5.43 × 10^2^	−89.7 ± 22.3	5.00 × 10^−3^
0.17	13.5 ± 1.45	2.47 × 10^3^ ± 6.12 × 10^2^	−97.7 ± 26.0	7.00 × 10^−3^
0.60	13.7 ± 1.30	2.57 × 10^3^ ± 5.58 × 10^2^	−93.7 ± 23.3	5.00 × 10^−3^
1.23	14.0 ± 1.42	2.70 × 10^3^ ± 6.25 × 10^2^	−88.0 ± 25.5	6.00 × 10^−3^
All Exp. data	13.8 ± 5.30·10^−1^	2.60 × 10^3^ ± 2.30 × 10^2^	−92.3 ± 9.55	2.40 × 10^−2^

**Table 8:** Parameter fitting to all experimental CuSO_4_·5H_2_O vapor pressure data for Equation (4).

Parameters	*A(m)*	*A(m)* − *B(m)*	*A(m)* − *B(m)* − *C(m)*
*A_0_*	5.02 ± 5.25 × 10^−1^	5.00 ± 5.60 × 10^−1^	5.68 ± 3.12
*A_1_*	−1.6 × 10^−3^ ± 3.50 × 10^−2^	2.36 × 10^−1^ ± 1.34	−7.94 ± 33.2
*A_2_*	3.20 × 10^−3^ ± 7.30 × 10^−2^	−4.70 × 10^−1^ ± 2.78	16.1 ± 36.8
*A_3_*	−1.60 × 10^−3^ ± 3.80 × 10^−2^	2.32 × 10^−1^ ± 1.45	−8.04 ± 35.9
*B_0_*	−3.31 × 10^2^ ± 3.24 × 10^2^	−3.25 × 10^2^ ± 3.39 × 10^2^	−7.44 × 10^2^ ± 1.92 × 10^3^
*B_1_*		−75.1 ± 4.23 × 10^2^	4.98 × 10^3^ ± 1.06 × 10^4^
*B_2_*		1.50 × 10^2^ ± 8.79 × 10^2^	−1.01 × 10^4^ ± 4.26 × 10^4^
*B_3_*		−74.0 ± 4.58 × 10^2^	5.04 × 10^3^ ± 1.21 × 10^4^
*C_0_*	−3.04 × 10^5^ ± 5.00 × 10^4^	−3.04 × 10^5^ ± 5.20 × 10^4^	−.39 × 10^5^ ± 2.97 × 10^5^
*C_1_*			−7.80 × 10^5^ ± 3.17 × 10^6^
*C_2_*			1.58 × 10^6^ ± 3.42 × 10^6^
*C_3_*			−7.89 × 10^5^ ± 3.42 × 10^6^
Residuals	2.62 × 10^−2^	2.59 × 10^−2^	2.56 × 10^−2^

**Table 9:** Comparison of evaporation cycles between NaCl and CuSO_4_·5H_2_O solutions using different fabrics.

Fabrics	NaCl (kg·m^−2^·h^−1^)		CuSO_4_·5H_2_O (kg·m^−2^·h^−1^)		H_2_O (kg·m^−2^·h^−1^)
	1C	15C	1C	15C	
PES	8.40 × 10^−2^	1.97 × 10^−1^	1.79 × 10^−1^	1.43 × 10^−1^	1.29 × 10^−1^
LPLA	7.30 × 10^−2^	1.42 × 10^−1^	1.33 × 10^−1^	1.34 × 10^−1^	1.43 × 10^−1^
Jut	8.90 × 10^−2^	2.04 × 10^−1^	1.43 × 10^−1^	1.26 × 10^−1^	1.50 × 10^−1^
Bam	8.60 × 10^−2^	9.70 × 10^−2^	2.34 × 10^−1^	9.10 × 10^−2^	1.89 × 10^−1^
Ara	1.30 × 10^−1^	1.66 × 10^−1^	1.95 × 10^−1^	1.80 × 10^−1^	1.71 × 10^−1^
WL-T	8.90 × 10^−2^	3.90 × 10^−2^	9.10 × 10^−2^	1.40 × 10^−1^	1.68 × 10^−1^

**Table 10:** Evaporation determinant factors in fabrics.

Determinant Factor	Effect on Evaporation
Temperature	Higher temperature: increases evaporation rate
Relative humidity	Increases relative humidity: decreases evaporation
Salt to treat	The crystal formation can enhance the evaporation
Solution	Vapor pressure affects the evaporation
Grammage	High grammage increases liquid absorption but reduces evaporation
Thickness	Less liquid retention favors lower salt precipitation
Porosity	A more porous fabric will make it more difficult for liquid to escape from inside the fabric and evaporate.

## Data Availability

The data presented in this study are available upon request from the corresponding author.
